# Prevalence and associated factors of intestinal parasitic infections among HIV clients attending Masaka Regional Referral Hospital, Uganda

**DOI:** 10.11604/pamj.2022.43.122.15957

**Published:** 2022-11-04

**Authors:** Sarah Mwebaza, Benedict Senyonga, Christine Atuhairwe, Ivan Mugisha Taremwa

**Affiliations:** 1 Institute of Allied Health Sciences, Clarke International University (Formerly, International Health Sciences University), Kampala, Uganda

**Keywords:** Intestinal parasites, HIV, opportunistic infection, Uganda

## Abstract

**Introduction:**

infection with Human Immune deficiency Virus (HIV) increases the risk of opportunistic infections, which aggravates life-long complications. We report the prevalence and the associated factors of intestinal parasites among HIV infected clients attending anti-retroviral therapy (ART) clinic at Masaka Regional Referral Hospital, in Uganda.

**Methods:**

this was a cross-sectional study that purposefully enrolled 410 HIV infected clients. Stool samples were macroscopically assessed, and analyzed using wet preparations, Formol ether concentration and Modified Ziehl Neelsen (ZN) techniques to identify cysts and ova of intestinal parasites. Further, a questionnaire was used to obtain data on socio-demographic, hygiene and immunologic markers. Logistic regression analysis was used to determine the associated factors of intestinal parasitic infection.

**Results:**

of the 410 adult HIV sero-positive clients enrolled, 58.0% (238/410) were females. Participants mean age was 26.8 years, (range of 18-59 years). The prevalence of intestinal parasites was 49/410 (11.95%; 95% confidence interval: 10.3 - 14.7). Intestinal parasites isolated were Giardia lamblia (N=10, 20.4%), strongloides stercolaris (N=4, 8.2%), and modified ZN showed Cryptosporidium species (N=35, 71.4%). Hand washing, history of not deworming in the previous 1 year, deteriorating HIV clinical stage and unprotected open water sources were the associated factors.

**Conclusion:**

this study reports a high prevalence of opportunistic intestinal parasites. As these are neglected tropical infections, early detection and exploration of the associated factors is key to their proper management.

## Introduction

Globally, up to 36.7 million people were living with human immune deficiency virus (HIV), and these risked suffering from Acquired Immune Deficiency Syndrome (AIDS) as the infection progresses due to opportunistic infections (OIs) [1,2]. There are various OIs among people living with HIV/AIDS, presenting as parasitic, bacterial and viral infections [3]. These take advantage of the delayed diagnosis, and failure to reach an immune status able to combat the disease, mainly due to adherence factors. Remarkably, nearly 100% of people infected with HIV in the developing countries suffer from diarrheal related illnesses, and 90% of these are due to intestinal parasites [4]. In Africa, the epidemiological burden of intestinal parasites among HIV clients remains high; for example, it was reported at 35.9% in Ethiopia [5], 11.4% in Nigeria [6], 50.9% in Kenya [7], 57.48% in Cameroon [8] and 65.3% in Burkina Faso [9]. As these are neglected tropical infections, they pause clinical and public health concerns that may exacerbate deaths if not diagnosed and treated early [10]. This study reports the prevalence of intestinal parasites, and the associated factors among HIV clients receiving anti-retro viral therapy at Masaka Regional Referral Hospital, in Uganda.

## Methods

**Study design, duration and setting:** this was a cross-sectional study, conducted at the immune suppressed syndrome (ISS) clinic of Masaka Regional Referral Hospital (MRRH) during the months of May to November 2017. Masaka Regional Referral Hospital (MRRH) is a 330 bed capacity, with an annual admission of 23,456 patients. The hospital´s catchment is spread across eight districts of Masaka, Rakai, Lyantonde, Lwengo, Ssembabule, Bukomansimbi, Kalungu and Kalagala. The ISS clinic started in 2001 as an auxiliary to the hospital to offer specialized services to the management of HIV clients, and has an average of 180 clients daily.

**Participants:** the study enrolled HIV-infected clients aged 18 years and above.

**Variables:** the dependent variables were socio-demographic characteristics, immune status as defined by the CD4 cell counts and WHO stage, deworming history, water source, and hygiene practices. The independent variable was the presence/absence of intestinal parasites among HIV clients.

**Data sources/measurements:** data were obtained using a questionnaire and laboratory analyses of stool specimens. A pre-tested questionnaire that centered on the socio-demographic and hygiene practices was administered to each participant. In addition, immunological values of their CD4-cell marker on that day were recorded, and clinical information on ART adherence was obtained from the participant´s file for the study analysis.

**Sample collection:** fresh stool was collected from each participant into a wide mouth stool container. Part of the sample was preserved in 10% formalin, and later on processed.

**Laboratory sample analyses:** samples were macroscopically observed for diagnostic features like consistency, worms or their segments. Wet preparations using physiologic saline were prepared and examined immediately for parasites eggs and cysts. In this, about one gram of the sample was transferred to a glass slide and emulsified into normal saline; a cover slip was applied on top and examined under X10 and X40 objective. If the cyst was seen, Dobell´s iodine was used to aid its identification [11]. These were supplemented by examination of a Formol-ether concentrated preparation [12], and Modified Ziehl-Neelsen (ZN) to detect the coccidian species [13].

**Bias and quality control:** this study did not carry any bias. As it involved laboratory investigation, we ensured quality control measures to guide the credibility of results. In this, we included a positive slide to quality control the performance of the ZN reagents. All positive samples were cross-examined and validated by a senior personnel attached to this laboratory. The investigators used standardized interpretation chats to identify and interpret the features of an organism [14].

**Data analysis:** data was entered in a statistical software package of EXCEL 5.0, (Microsoft, and Redmond, WA, USA) and transferred to STATA 12 (College Station, TX, USA) for analysis. Mean median, inter-quartile range and standard deviation were used. Univariate and multivariate logistic regression analyses were used to look for statistical significance with associated factors of intestinal parasitic infection. This was based on those variables with P-value of 0.05 or less in the univariate logistic regression and was considered for the multivariable logistic regression analysis. Variables with p-value <0.05 were considered statistically significant.

**Ethical approval:** ethical approval was obtained from the research and ethics committee of Clarke International University (Formerly, International Health Science University: UG-REC-015, reference number: IHSU-REC/0083). We obtained written informed consent from each participant, and ensured confidentiality. Results were availed to the attending doctor for participant's care.

## Results

**Socio-demographics and clinical characteristics:** a total of 410 adults were recruited. Of these, 58.0% (238/410) were females; their mean age was 26.8 years, (range of 18-59 years) and 34.4% were in clinical stage IV, as indicated in Table 1.

**Table 1:** demographic and clinical characteristics of study participants

Characteristic	Frequency (n)	Percentage (%)
**Age (years)**		
< 20	73	17.8
20 to 24	69	16.8
25 to 29	78	19.0
30 to 34	93	22.7
35 to 39	54	13.2
40 to 44	29	7.1
45 and above	13	3.2
**Gender**		
Males	172	42.0
Females	238	58.0
**Marital status**		
Single	48	11.7
Married	197	48.0
Separated	87	21.2
Divorced	51	12.4
Widowed	27	6.6
**WHO clinical staging**		
Stage I	108	26.3
Stage II	97	23.7
Stage III	64	15.6
Stage IV	141	34.4

**Prevalence of intestinal parasites among the HIV clients:** the prevalence of intestinal parasites was 11.95% (95% confidence interval: 10.3 - 14.7). Of the 49 parasites isolated, *Giardia lamblia* accounted for 10 (20.4%), *Strongloides stercolaris* 4 (8.2%), and Modified ZN showed 35 (71.4%) positives with *Cryptosporidium species*.

**Factors associated with intestinal parasite infestation among HIV clients:** univariate logistic regression analysis of the socio-demographic, hygiene and immunological factors was done as given in Table 2. Variables that had a statistical significance defined by a p-value <0.05 were fitted in a logistic regression model. Accordingly, the factors associated with intestinal parasitic infections were: handwashing, history of not deforming in the previous 1 year, deteriorating clinical stage and unprotected open water sources, as given in Table 3.

**Table 2:** univariate logistic regression analysis of the predisposing factors of acquiring intestinal parasites

Variable	Categories	P-value	Crude Odds Ratio (95% Confidence Interval)
Hand washing	No Yes	0.002	3.972 (2.016-7.436)
CD4 (Cells/mm3)	1-100	0.001	6.818 (4.917-9.003)
	201-200		
	201-300		
	301-400		
	401-500		
	>500		
Deworming history	No Yes	0.003	5.750 (4.907-8.577)
Marital status	Divorced		
	Married		
	Widowed		
	Single	0.09	7.811 (5.998-8.109)
Education level	None		13.1 (10.367-18.439)
	Primary	0.849	1.467 (0.951-2.890)
	Secondery		
	Tertiary		
Occupation	Agriculture	0.059	0.997 (0.988-2.167)
	Business		
	NGO		
	Self-employed		
	Government		
	Unemployed		
Water source	Spring		
	Tap		
	Well	0.000	7.129 (5.173-9.932)
	Borehole		
Use of pitlatrin	No Yes	0.319	1.699 (1.098-2.964)

* denotes statistical significance at p<0.05

**Table 3:** multivariate logistic regression analysis of the associated factors of acquiring intestinal parasitic infections

Variable	Categories	P-value	Adjusted Odds Ratio (95% Confidence Interval)
Hand washing	No Yes	0.000*	3.633 (1.784-6.112)
CD4 (Cells/mm^3^)	1-100	0.003*	6.561 (3.704-8.176)
	201-200		
	201-300		
	301-400		
	401-500		
	>500		
Deworming history	No Yes	0.001*	5.481 (4.397-7.168)
Water source	Spring		
	Tap		
	Well	0.000*	6.471 (5.813-8.644)

* denotes statistical significance at p<0.05

## Discussion

The prevalence of intestinal parasites among HIV clients at Masaka Regional Referral Hospital was found to be 11.95%, a value that is less than 35.9% that was reported in Ethiopia [5], 11.4% in Nigeria [6], 50.9% in Kenya [7], 57.48% in Cameroon [8] and 65.3% in Burkina Faso [9]. The observed variation is ascribed to the fact that participants in our study were on anti-retroviral therapy (ART), with the implementation of ‘test and treat´ policy which reduces the risk of opportunistic infections [15]. The intestinal parasites detected were *Gardia lamblia* (N=10), *Strongloides stercolaris* (N=4) and *Cryptosporidium species* (N=35). The observed distribution agrees with a systematic review which revealed that *Cryptosporidium* species accounts for the majority of the infections in HIV clients [16], and a study in Kenya indicated a similar trend of infection [17]. Also, studies in India reported *Cryptosporidium* as the most prevalent intestinal parasite in HIV [18,19]. Although complex, our study found a significant proportion (7.3%) of ART defaulters, which may account for the high prevalence reported.

As HIV/AIDS is associated with deranged immunologic status, participants with a lowered CD4 cell count (below 500 cells/μL of blood) were almost 7 times (6.561) compared to those whose immunity had greater CD4 cell counts (> 500 cells/μL of blood). Participants who had not been dewormed in the previous 1 year were 6 times (OR=5.481); those who did not wash their hands before eating and after using latrines (OR=3.633) as well as those who used unprotected open water sources (OR=6.471) were more likely to have intestinal parasites. The study findings are in agreement with related studies which indicated that poor personal hygiene, failure to deworm and deranged immunological factors can precipitate opportunistic infections in HIV leading to morbidity and mortality [20,21]. Our results should be interpreted in light of the fact that we examined a single stool sample, which may limit the benefits of improving the detection rate. Also, non-parasitic causes of diarrhea like viral and bacterial agents were not detected. Although modified ZN technique was used for *Cryptosporidium* species, it is less sensitive as compared to methods like polymerase chain reaction and direct fluorescent-antibody tests; as such, the prevalence of cryptosporidiosis could have been under estimated.

## Conclusion

The study reports a high prevalence of intestinal parasites, mainly due to *Cryptosporidium species, Stronglyloides stercolaris* and *Gardia lamblia*. The associated factors to the acquisition of parasitic infections were CD4 cell counts below 100 cells per microliter of blood, not washing hands, not being dewormed in the previous 1 year and using water from contaminated open sources particularly, the wells.

### What is known about this topic

- Majority of the individuals living with HIV suffer from severe diarrheal related illnesses, that results into morbidity, which if not controlled can cause death;
- The gastrointestinal parasites are of clinical and public concern due to their association with poor hygiene and living conditions seen in low income countries;
- The effect of intestinal parasites is key; considering the harm caused to the affected individual and the urgency of which a clinical intervention is required.

### What this study adds

- The study found a high prevalence of intestinal parasites among people living with HIV, yet stool examination is not routinely performed;
- The isolated intestinal parasites are: Giardia lamblia, Strongloides stercolaris, and Cryptosporidium species;
- The associated factors of intestinal parasitic infection were: poor hygiene practices, irregular deworming, and inadequate water sanitation.
